# Erythropoietin monotherapy for neuroprotection after neonatal encephalopathy in low-to-middle income countries: a systematic review and meta-analysis

**DOI:** 10.1038/s41372-021-01132-4

**Published:** 2021-06-26

**Authors:** Phoebe Ivain, Paolo Montaldo, Aamir Khan, Ramyia Elagovan, Constance Burgod, Maria Moreno Morales, Stuti Pant, Sudhin Thayyil

**Affiliations:** 1 Centre for Perinatal Neuroscience, Imperial College London, London, UK; 2 Department of Neonatal Intensive Care, Universita degli Studi della Campania Luigi Vanvitelli, Naples, Italy

**Keywords:** Brain injuries, Outcomes research

## Abstract

**Objective:**

We examined whether erythropoietin monotherapy improves neurodevelopmental outcomes in near-term and term infants with neonatal encephalopathy (NE) in low-middle income countries (LMICs).

**Methods:**

We searched Pubmed, Embase, and Web of Science databases to identify studies that used erythropoietin (1500–12,500 units/kg/dose) or a derivative to treat NE.

**Results:**

Five studies, with a total of 348 infants in LMICs, were retrieved. However, only three of the five studies met the primary outcome of death or neuro-disability at 18 months of age or later. Erythropoietin reduced the risk of death (during the neonatal period and at follow-up) or neuro-disability at 18 months or later (*p* < 0.05). Death or neuro-disability occurred in 27.6% of the erythropoietin group and 49.7% of the comparison group (risk ratio 0.56 (95% CI: 0.42–0.75)).

**Conclusion:**

The pooled data suggest that erythropoietin monotherapy may improve outcomes after NE in LMICs where therapeutic hypothermia is not available.

## Introduction

Neonatal encephalopathy (NE) occurs in 1–4 per 1000 live births in high-income countries and is estimated to be as high as 26 per 1000 live births in low and middle-income countries (LMICs) [1, 2]. Of the annual 1 million neonatal deaths attributed to NE globally, 99% occur in LMICs [1, 3, 4].

Therapeutic hypothermia (TH) is the standard of care for newborn babies with NE in high-income countries, with randomized-controlled trials demonstrating the ability of TH to improve outcomes for babies with NE in these settings [5]. However, the safety and efficacy of cooling in LMIC populations are still unclear. A systematic review of cooling trials in LMICs did not report any significant benefit of cooling therapy for NE babies [6]. Furthermore, cooling may lack applicability in LMICs due to a shortage of neonatal intensive care units and the necessary transport needed to administer the therapy within the 6-h window [7].

Given these challenges, an alternative neuroprotective approach is vital in LMICs [8]. Although many novel neuroprotective strategies have been investigated in pre-clinical models (melatonin, N-acetyl cysteine, stem cells), erythropoietin is currently the most promising agent due to its dual neuroprotective and regenerative properties, and a wide therapeutic window of up to 24 h [9–11]. When used as a monotherapy, erythropoietin promotes neuro-regeneration in pre-clinical models of hypoxic-ischaemic encephalopathy [2, 12–14]. There are also a number of ongoing trials assessing the combined effectiveness of erythropoietin alongside cooling therapy in infants with moderate-severe encephalopathy in high-income countries [2, 15]. However, these trials will not provide information regarding the safety and efficacy of erythropoietin without adjunct cooling therapy in LMICs.

Therefore, we systematically reviewed the published literature to examine whether erythropoietin monotherapy improves neurodevelopmental outcomes in near-term and term infants with NE in LMICs.

## Methods

### Search strategy

We used the Cochrane Handbook of Systematic Review of Interventions methodology for literature search, data extraction, quality assessment, and meta-analysis [16]. We searched Pubmed, Embase, and Web of Science from 1 January 1999 to 1 June 2020, using the following MeSH terms: [hypoxic ischaemic encephalopathy OR NE] AND [erythropoietin] AND [newborn] AND [clinical trials]. We excluded abstracts for which the full text was unavailable or in a language other than English. We also examined the reference lists of the retrieved articles and review papers to identify any studies missed by the initial search. No method restrictions were set and only studies from LMICs were deemed eligible for inclusion in the meta-analysis. LMICs were defined according to the World Bank classifications by income level (2019–2020).

All titles and abstracts identified as potentially relevant were assessed. Two reviewers (PI and AK) independently selected the studies to be included, extracted the data, and assessed the quality based on allocation concealment, blinding of outcome assessment, adherence to intention to treat analysis, and completeness and quality of follow-up. Duplicated studies were removed from search results. Discrepancies in reviewer selections were resolved by a third reviewer (PM). The data for all included studies was collected using a formatted spreadsheet according to the “PICO” format (patient, intervention, comparison, and outcome) of the published results.

### Study design and inclusion criteria

We included randomized-control trials as well as all non-randomized and case-control studies in which parenteral (intravenous or subcutaneous) erythropoietin or one of its analogues was administered within one week of postnatal life and compared with placebo or usual care in patients with NE. The study population included only neonates born at ≥36 weeks gestation. The definition of asphyxia used in high-income countries includes investigations that are not widely available in LMICs (such as amplitude-integrated electroencephalogram).

Furthermore, Apgar scores are often not available or not collected beyond 5 min of birth, especially when babies are born at home. Therefore, we decided to use the same definition of asphyxia as in the hypothermia for encephalopathy in low- and middle-income countries (HELIX) Trial [7]. Asphyxia was considered if at least one of the following criteria was met: (i) Apgar score ≤5 at 5 min, (ii) cord or arterial blood pH ≤ 7.0, (iii) base deficit >12 mmol/L within the first hour after birth, or (iv) ongoing resuscitation or mechanical ventilation at 5 min of life. NE was defined using a detailed neurological examination performed prior to enrolment which was assessed against objective criteria.

### Outcome measures

The primary outcome was a composite measure of mortality or neuro-disability at 18 months of age or later. The secondary outcome measures were mortality, cerebral palsy, brain injury on conventional magnetic resonance imaging, moderate-to-severe cognitive impairment, and any adverse outcomes as a result of erythropoietin administration.

Adverse outcomes included: persistent hypotension, grade IV intraventricular haemorrhage on ultrasound, pulmonary haemorrhage, persistent pulmonary hypertension, systematic hypertension, major venous or arterial thrombosis, prolonged blood coagulation, polycythaemia, culture-proven sepsis, necrotising enterocolitis, cardiac arrhythmia requiring therapy, severe thrombocytopenia (platelet count <25,000 per mL), persistent metabolic acidosis lasting over 12 h after birth, renal failure (anuria > 48 h with azotaemia), pneumonia, subcutaneous fat necrosis, and neurological examination at discharge.

### Analysis and quality assessment

We used a random-effects model for the meta-analysis (RevMan Version 5.4, Cochrane Collaboration, 2014). Risk ratios were also calculated for available primary and secondary outcomes and reported with 95% confidence intervals (CI). Statistical heterogeneity was examined using the I^2^ index.

The risk of bias for each study was determined based on statements relating to random sequence generation, allocation concealment, blinding of participants and personnel, outcome assessment, incomplete outcome data, and selective reporting. Each property was ranked as “low risk”, “unclear risk”, or “high risk” according to the quality assessment of diagnostic accuracy studies (QUADAS 2) tool and is presented visually as a graph and summary figure.

## Results

**Fig. 1:**
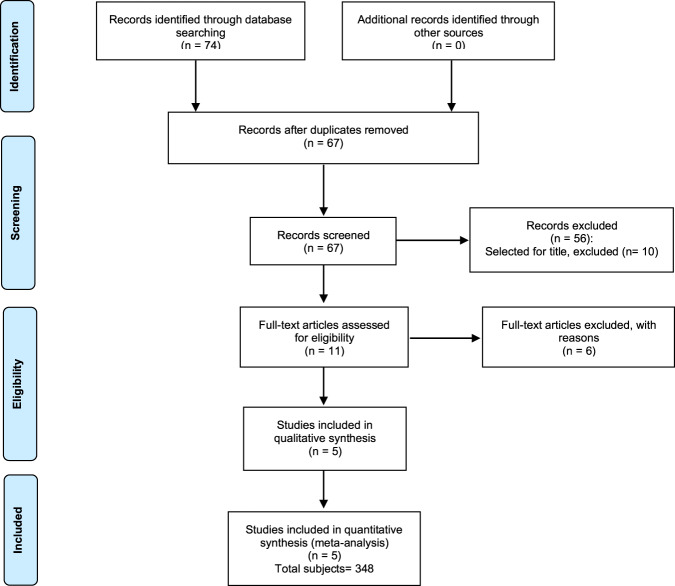
Flow chart of the literature search. The diagram shows the different phases of the systematic review.

Our search strategy yielded a total of 74 potentially relevant studies. Eleven studies met the initial search criteria, of which five were selected for inclusion (shown in Fig. 1, Table 1) [17–21]. The excluded studies are listed in Table 2 [22–27].

**Table 1:** Studies included in the meta-analysis.

Study (year)	*N*	Country of study	Study Population	Neurological assessment	Intervention	Comparison	Primary outcome measure
Avasiloaiei (2013) [17]	67	Romania	≥37–40 weeks with perinatal asphyxia	American Academy of Paediatrics and American College of Obstetricians and Gynaecologists 2003, 3 of 4 criteria met to be included	SC erythropoietin (1000 IU/kg) once a day for the first 3 days	Routine intensive care OR IV Phenobarbital (40 mg/kg) as a single dose in the first 4 h after birth	Death or Disability at 18 months using Bayley Infant Scales of Development II
Elmahdy (2010) [20]	30	Egypt	≥38–42 weeks with mild or moderate NE	Sarnat and Sarnat 1976, >3 signs in one of the following categories: Normal, Mild NE, Moderate NE, or Severe NE	SC erythropoietin (2500 IU/kg) once a day for the first 5 days	Routine intensive care	Death or Disability at 6 months using Denver Developmental Screening Test II
El Shimi (2013) [18]	20	Egypt	≥37–40 weeks with perinatal asphyxia	Sarnat and Sarnat 1976, >3 signs in one of the following categories: normal, Mild NE, moderate NE, or severe NE	SC erythropoietin (1500 IU/kg) as a single dose	Routine intensive care	Death or Disability at 3 months using neuromuscular function scale (NMS)
Malla (2017) [19]	100	India	≥37–40 weeks, <6 h age, AND moderate or severe NE	Sarnat and Sarnat 1976, >3 signs in one of the following categories: Normal, Mild NE, Moderate NE, or Severe NE	IV erythropoietin (500 IU/kg) on D1,3,5	Placebo	Death or moderate or severe disability at 19 months using Bayley Infant Scales of Development II
Zhu (2009) [21]	153	China	≥37–40 weeks AND clinical evidence of moderate or severe NE	Sarnat and Sarnat 1976, >3 signs in one of the following categories: Normal, Mild NE, Moderate NE, or Severe NE	Forty-five had SC/IV erythropoietin (300 IU/kg) and 28 had SC/IV erythropoietin (500 IU/kg) on alternate days for 2 weeks	Routine intensive care	Death or disability at 18 months using Bayley Infant Scales of Development II; the presence of cerebral palsy and Mental Development Index (MDI) < 70

**Table 2:** Studies excluded from the meta-analysis.

Study (year)	*N*	Country of Study	Study population	Intervention	Comparison	Primary outcome measure	Reason for exclusion
Wang (2011) [25]	62	China	≥36 weeks with moderate or severe NE	Erythropoietin IV 200IU/kg × 3 doses per week for 2–4 weeks	Placebo or healthy infants	Death or disability at 6 months	No full-text available in English
Lv (2017) [26]	41	China	≥37 weeks with moderate or severe NE	Erythropoietin 200IU/kg for 10 days and TH 72 h	TH alone	Death or disability at 9 months	No extractable outcome data and therapeutic hypothermia used
Wu (2016) [22]	50	USA	≥36 weeks with moderate or severe NE	TH and erythropoietin IV 1000IU/kg × 5 days	TH alone	Death or disability at 12 months	Conducted in the USA and therapeutic hypothermia used
Baserga (2015) [24]	30	USA	≥36 weeks with moderate or severe HIE	Darbepoetin 2 or 10 ug/kg × 2 doses within 12 h birth and at 7 days	Placebo	Death or adverse events at 1-month	Conducted in USA, outcome at only at 1-month postnatal age, and therapeutic hypothermia used
Rogers (2014) [23]	24	USA	≥36 weeks with mild-to-severe HIE	Erythropoietin 250, 500, 1000, or 2500 U/kg every 48 h × 6 doses	N/A	Death or disability at 8–34 months	Conducted in the USA, no control group, and therapeutic hypothermia used
Valera (2015) [27]	15	Spain	≥36 weeks with HIE	Erythropoietin at 400 U/kg every 48 h for 2 weeks	N/A	Death or disability at 18 months	No control group and therapeutic hypothermia used

All five studies included in our analysis were performed in hospitals within LMICs and reported post-natal neurodevelopmental outcomes. Data were extracted for a total of 348 babies with NE from four different countries. Three studies compared erythropoietin to routine neonatal intensive care and one study compared it to a placebo. Another study compared erythropoietin to both routine care and TH, although the babies who received TH were not included in our analysis to allow for comparison with the other included studies. Furthermore, no adverse effects specific to erythropoietin administration were found.

**Fig. 2:**
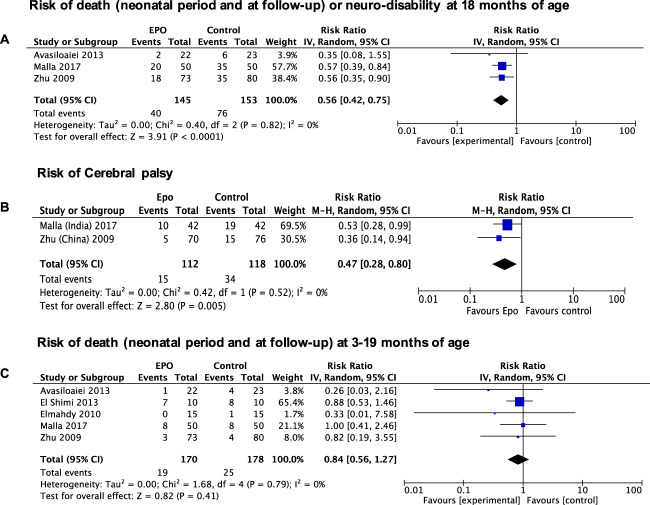
Forest plot of the observed effect of erythropoietin in the included studies. Effect of erythropoietin on (**A**) death (neonatal period and at follow-up) or neuro-disability at 18 months of age or later, (**B**) cerebral palsy, and (**C**) death during the neonatal period and at follow-up (between three and 19 months of age) in infants with NE.

Three of the five included studies reported outcome at 18 months of age or later. Erythropoietin significantly reduced the risk of death or neuro-disability in these three studies (*p* < 0.05). Death or neuro-disability occurred in 27.6% of the erythropoietin group and 49.7% of the comparison group (risk ratio 0.56 (95% CI: 0.42–0.75)) (shown in Fig. 2a).

The secondary outcome of cerebral palsy was reported in two of the five included studies. Erythropoietin administration also significantly reduced the risk of cerebral palsy (*p* < 0.05). Cerebral palsy was reported in 13.4% of the erythropoietin group and 22.8% of the control group (risk ratio 0.47 (95% CI: 0.28–0.80)) (shown in Fig. 2b). However, the pooled data showed no difference in neonatal mortality or death reported at follow-up between three and 19 months of age between the erythropoietin and comparison group infants (*p* = 0.41). The death occurred in 11.2% of the erythropoietin group and 14.0% of the comparison group (risk ratio 0.84 (95% CI: 0.56–1.27)) (shown in Fig. 2c). Meta-analyses were not conducted for the other secondary outcomes as they were either infrequently reported or, or in the case of brain injury, the scoring system was not specified.

The included studies showed no evidence of statistical heterogeneity (*I*^2^ = 0%; *p* > 0.05). This is most likely due to the included studies having a relatively small sample size. A funnel plot was not conducted due to the low number of included studies.

**Fig. 3:**
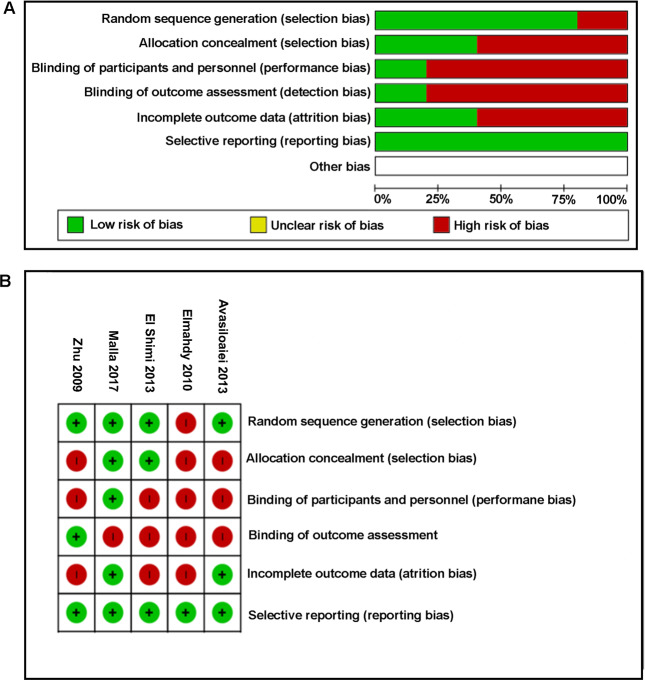
Risk of bias assessment. (**A**) Risk of bias graph and (**B**) risk of bias summary.

Each individual study was also assessed for risk of bias (shown in Fig. 3). Follow-up varied greatly between studies, with a range between three and 19 months of age. All but one study failed to state whether participants and personnel were blinded to a treatment group or outcome [19]. Regardless, clinical and assessment data from all participants were reported at follow-up in all studies.

## Discussion

In this systematic review and meta-analysis, we assessed five studies that reported the role of erythropoietin as a neuroprotective agent in term infants with NE in LMIC settings. Our analysis shows that erythropoietin significantly reduced the risk of death or neuro-disability and cerebral palsy in the three studies with outcome reported at 18 months of age. However, erythropoietin did not reduce the risk of neonatal mortality or death reported at follow-up between three and 19 months of age. Furthermore, there were no adverse effects specific to erythropoietin administration.

Our results are in accordance with previous meta-analyses of erythropoietin efficacy for infants with NE [28, 29]. One meta-analysis assessed six randomised-controlled trials, the other examined 15 studies comparing five interventions. Both reported that erythropoietin decreased the risk of mortality and neurodevelopmental delay at 18 months. However, these meta-analyses included studies from both LMIC and high-income countries and therefore did not account for efficacy and mechanistic differences between these populations [6]. In fact, the HELIX feasibility study, which examined the effectiveness of a servo-controlled cooling device in India, has highlighted significant differences in infants with NE in LMICs including lower birth weight and a high incidence of gastric bleeding [30].

The effectiveness of erythropoietin as a neuroprotective agent is hypothesised to be related to its anti-excitatory, anti-inflammatory and anti-oxidant properties [31]. One of the main actions of erythropoietin is to reduce free iron accumulation that occurs following secondary brain injury in NE [32]. By inducing erythropoiesis, erythropoietin, in turn, promotes neurogenesis and other regenerative processes [9, 14, 33, 34]. The compelling evidence from pre-clinical studies and trials in both low-middle and high-income countries suggests that erythropoietin monotherapy may be useful in LMIC clinical settings where TH is not compatible [2, 15].

One of the studies included in our analysis, El Shimi et al., compared erythropoietin with both supportive care and TH [18]. The study described individual statistics favouring TH for neuroprotection, but these were not statistically significant. As follow-up was only at three months of age, a later neurological assessment may provide considerably more information regarding neuroprotective differences between erythropoietin and TH use in NE babies. Overall, there is still a lack of evaluative data comparing varying dosages of erythropoietin with and without TH in LMIC settings, underpinning the need for more robust and definitive trials.

All the studies included in our meta-analysis used erythropoietin as monotherapy. However, different dosage regimens, routes of administration (intravenous or subcutaneous), and timings were used across the individual studies. Although a therapeutic dose in term newborns has yet to be determined, 300 and 500 IU/kg/dose has been shown to be most effective when administered over a longer period in preterm infants [35, 36]. Whereas the study by Malla et al. used a singular dose at 500 IU/kg on days 1, 3, and 5 [19], making it difficult to derive the true neuroprotective effect of erythropoietin in this instance. While randomised controlled trials using a dose of 1000 IU/kg/dose are ongoing in high-income countries, there is no such trial occurring in LMICs [15]. Therefore, forthcoming clinical trials examining erythropoietin use for neuroprotection in LMICs should focus on optimizing protocols using a higher dosage in larger study populations.

We also found significant heterogeneity in the patient populations of the included studies with regard to national economic status, inclusion criteria, and NE definitions. This reflects an extreme heterogeneity in the study design and a possible introduction of biases. An example of this being the use of Sarnat staging to classify NE in all but one of the included studies, whereas a different NE severity was set as an entry criterion [17]. Two studies included infants with moderate-to-severe NE [19, 21], while another only included infants with mild-to-moderate NE [20]. The other two studies used perinatal asphyxia as the entry criterion [17, 18].

The length of study duration also varied widely among the included studies (ranging from six to 41 months of patient recruitment), which may signify differences in clinical management over a longer period of time. In addition, a lack of secondary outcome data was noted, as only two studies reported results for cerebral palsy. Furthermore, only three of the studies reported outcome at 18 months or later, indicating inadequate follow-up of NE patients. Longer recruitment timescales which yield small sample sizes and limited follow-up data suggest a lack of available resources for both study design and management in those clinical settings.

While the data reported in this meta-analysis shows the beneficial effect of erythropoietin, there are some limitations. Firstly, the cumulative sample sizes of all included studies were small, with one study, not randomising recruits and four studies not blinded. Secondly, there was a great amount of heterogeneity among the included studies, which was accounted for by using a random effects model. Also, the age interval at the time of neurodevelopmental outcome was wide, with the earliest reported outcome at three months of age and the latest at 19 months. Despite this variation in age at follow-up, three of the five studies reported outcomes at greater than 18 months of age and used the same neurological examination (Bayley Scales of Infant Development Volume III) to evaluate participants.

## Conclusion

The pooled data from these small clinical trials suggest that erythropoietin may improve neurodevelopmental outcomes in neonates who have sustained NE within LMICs. Erythropoietin has shown a beneficial effect when used as a monotherapy and demonstrated safety and efficacy when used at a variety of doses, with no adverse events. Further evaluation of erythropoietin in adequately powered clinical trials is necessary. In future trials, it will be crucial for researchers and clinicians in LMICs to collaborate with experts and form interest groups to clearly define appropriate guidelines, primary, and secondary outcomes for erythropoietin usage. Thus far, erythropoietin has shown promise as a neuroprotective agent and future implementation may greatly improve outcomes for NE infants with brain injury in LMICs.

## References

[1] Kurinczuk JJ, White-Koning M, Badawi N (2010). Epidemiology of neonatal encephalopathy and hypoxic-ischaemic encephalopathy. Early Hum Dev.

[2] Oorschot DE, Sizemore RJ, Amer AR. Treatment of neonatal hypoxic-ischemic encephalopathy with erythropoietin alone, and erythropoietin combined with hypothermia: history, current status, and future research. Int J Mol Sci. 2020;21. 10.3390/ijms21041487 PMC7073127 32098276

[3] Lee AC, Kozuki N, Blencowe H, Vos T, Bahalim A, Darmstadt GL (2013). Intrapartum-related neonatal encephalopathy incidence and impairment at regional and global levels for 2010 with trends from 1990. Pediatr Res.

[4] Lawn JE, Cousens S, Zupan J (2005). Lancet neonatal survival steering T. 4 million neonatal deaths: when? Where? Why?. Lancet.

[5] Jacobs SE, Berg M, Hunt R, Tarnow-Mordi WO, Inder TE, Davis PG (2013). Cooling for newborns with hypoxic ischaemic encephalopathy. Cochrane Database Syst Rev.

[6] Pauliah SS, Shankaran S, Wade A, Cady EB, Thayyil S (2013). Therapeutic hypothermia for neonatal encephalopathy in low- and middle-income countries: a systematic review and meta-analysis. PLoS ONE.

[7] Thayyil S, Oliveira V, Lally PJ, Swamy R, Bassett P, Chandrasekaran M (2017). Hypothermia for encephalopathy in low and middle-income countries (HELIX): study protocol for a randomised controlled trial. Trials.

[8] Montaldo P, Pauliah SS, Lally PJ, Olson L, Thayyil S (2015). Cooling in a low-resource environment: lost in translation. Semin Fetal Neonatal Med.

[9] Juul SE, Pet GC (2015). Erythropoietin and neonatal neuroprotection. Clin Perinatol.

[10] Wu YW, Bauer LA, Ballard RA, Ferriero DM, Glidden DV, Mayock DE (2012). Erythropoietin for neuroprotection in neonatal encephalopathy: safety and pharmacokinetics. Pediatrics.

[11] Iwai M, Stetler RA, Xing J, Hu X, Gao Y, Zhang W (2010). Enhanced oligodendrogenesis and recovery of neurological function by erythropoietin after neonatal hypoxic/ischemic brain injury. Stroke.

[12] Kellert BA, McPherson RJ, Juul SE (2007). A comparison of high-dose recombinant erythropoietin treatment regimens in brain-injured neonatal rats. Pediatr Res.

[13] Iwai M, Cao G, Yin W, Stetler RA, Liu J, Chen J (2007). Erythropoietin promotes neuronal replacement through revascularization and neurogenesis after neonatal hypoxia/ischemia in rats. Stroke.

[14] Wang L, Zhang Z, Wang Y, Zhang R, Chopp M (2004). Treatment of stroke with erythropoietin enhances neurogenesis and angiogenesis and improves neurological function in rats. Stroke.

[15] Juul SE, Comstock BA, Heagerty PJ, Mayock DE, Goodman AM, Hauge S (2018). High-dose erythropoietin for asphyxia and encephalopathy (HEAL): a randomized controlled trial-background, aims, and study protocol. Neonatology.

[16] Cumpston M, Li T, Page MJ, Chandler J, Welch VA, Higgins JP (2019). Updated guidance for trusted systematic reviews: a new edition of the cochrane handbook for systematic reviews of interventions. Cochrane Database Syst Rev.

[17] Avasiloaiei A, Dimitriu C, Moscalu M, Paduraru L, Stamatin M (2013). High-dose phenobarbital or erythropoietin for the treatment of perinatal asphyxia in term newborns. Pediatr Int.

[18] El Shimi MS, Awad HA, Hassanein SM, Gad GI, Imam SS, Shaaban HA (2014). Single dose recombinant erythropoietin versus moderate hypothermia for neonatal hypoxic ischemic encephalopathy in low resource settings. J Matern Fetal Neonatal Med.

[19] Malla RR, Asimi R, Teli MA, Shaheen F, Bhat MA (2017). Erythropoietin monotherapy in perinatal asphyxia with moderate to severe encephalopathy: a randomized placebo-controlled trial. J Perinatol.

[20] Elmahdy H, El-Mashad AR, El-Bahrawy H, El-Gohary T, El-Barbary A, Aly H (2010). Human recombinant erythropoietin in asphyxia neonatorum: pilot trial. Pediatrics.

[21] Zhu C, Kang W, Xu F, Cheng X, Zhang Z, Jia L (2009). Erythropoietin improved neurologic outcomes in newborns with hypoxic-ischemic encephalopathy. Pediatrics.

[22] Wu YW, Mathur AM, Chang T, McKinstry RC, Mulkey SB, Mayock DE, et al. High-dose erythropoietin and hypothermia for hypoxic-ischemic encephalopathy: a phase II trial. Pediatrics. 2016;137. 10.1542/peds.2016-0191 27244862

[23] Rogers EE, Bonifacio SL, Glass HC, Juul SE, Chang T, Mayock DE (2014). Erythropoietin and hypothermia for hypoxic-ischemic encephalopathy. Pediatr Neurol.

[24] Baserga MC, Beachy JC, Roberts JK, Ward RM, DiGeronimo RJ, Walsh WF (2015). Darbepoetin administration to neonates undergoing cooling for encephalopathy: a safety and pharmacokinetic trial. Pediatr Res.

[25] Wang YJ, Pan KL, Zhao XL, Qiang H, Cheng SQ (2011). Therapeutic effects of erythropoietin on hypoxic-ischemic encephalopathy in neonates. Zhongguo Dang Dai Er Ke Za Zhi.

[26] Lv HY, Wu SJ, Wang QL, Yang LH, Ren PS, Qiao BJ (2017). Effect of erythropoietin combined with hypothermia on serum tau protein levels and neurodevelopmental outcome in neonates with hypoxic-ischemic encephalopathy. Neural Regen Res.

[27] Valera IT, Vázquez MDC, González MDR, Jaraba MP, Benítez MVR, de la Cámara Moraño C (2015). Erythropoietin with hypothermia improves outcomes in neonatal hypoxic ischemic encephalopathy. J Clin Neonatol.

[28] Lee CYZ, Chakranon P, Lee SWH (2019). Comparative efficacy and safety of neuroprotective therapies for neonates with hypoxic ischemic encephalopathy: a network meta-analysis. Front Pharmacol.

[29] Razak A, Hussain A (2019). Erythropoietin in perinatal hypoxic-ischemic encephalopathy: a systematic review and meta-analysis. J Perinat Med.

[30] Oliveira V, Kumutha JR, N E, Somanna J, Benkappa N, Bandya P (2018). Hypothermia for encephalopathy in low-income and middle-income countries: feasibility of whole-body cooling using a low-cost servo-controlled device. BMJ Paediatr Open.

[31] Dame C, Juul SE, Christensen RD (2001). The biology of erythropoietin in the central nervous system and its neurotrophic and neuroprotective potential. Biol Neonate.

[32] Rathnasamy G, Ling EA, Kaur C (2011). Iron and iron regulatory proteins in amoeboid microglial cells are linked to oligodendrocyte death in hypoxic neonatal rat periventricular white matter through production of proinflammatory cytokines and reactive oxygen/nitrogen species. J Neurosci.

[33] Kumral A, Gonenc S, Acikgoz O, Sonmez A, Genc K, Yilmaz O (2005). Erythropoietin increases glutathione peroxidase enzyme activity and decreases lipid peroxidation levels in hypoxic-ischemic brain injury in neonatal rats. Biol Neonate.

[34] Sakanaka M, Wen TC, Matsuda S, Masuda S, Morishita E, Nagao M (1998). In vivo evidence that erythropoietin protects neurons from ischemic damage. Proc Natl Acad Sci USA.

[35] Song J, Sun H, Xu F, Kang W, Gao L, Guo J (2016). Recombinant human erythropoietin improves neurological outcomes in very preterm infants. Ann Neurol.

[36] Ohls RK, Kamath-Rayne BD, Christensen RD, Wiedmeier SE, Rosenberg A, Fuller J (2014). Cognitive outcomes of preterm infants randomized to darbepoetin, erythropoietin, or placebo. Pediatrics.

